# Using multileaf collimator interleaf leakage to extract absolute spatial information from electronic portal imaging device images

**DOI:** 10.1120/jacmp.v8i1.2226

**Published:** 2007-02-28

**Authors:** Zhanrong Gao, Janos Szanto, Lee Gerig

**Affiliations:** ^1^ Carleton University Department of Physics Ottawa Ontario Canada; ^2^ Ottawa Hospital Regional Cancer Centre Department of Medical Physics Ottawa Ontario Canada

**Keywords:** EPID, isocenter, Radon transform, orientation correction, mechanical consistency

## Abstract

Electronic portal imaging devices (EPIDs) are potentially valuable tools for linear accelerator quality assurance and for measuring and analyzing geometric variations in radiation treatment delivery. Geometric analysis is more robust if referenced against an absolute position such as the isocenter (collimator axis of rotation), allowing the observer to discriminate between various setup errors and jaw or multileaf collimator (MLC) calibration errors. Unfortunately, mechanical instabilities in EPIDs make such analysis difficult. In the present work, we describe how MLC interleaf radiation leakage, hidden in the background of portal images, can be extracted and analyzed to find the field isocenter perpendicular to leaf travel direction. The signal from the interleaf radiation leakage is extracted to provide a precise and accurate determination of the isocenter location in the direction perpendicular to MLC leaf travel. In the direction of leaf travel, the minimization of residuals between planned and measured leaf positions is used to determine the isocenter. This method assumes that leaf positioning errors are randomly distributed. The validity of the method for determining the angular deviation between EPID image grid lines and collimator angle and for determining the known isocenter position is experimentally established.

PACs numbers: 87.53.Oq, 87.53.Xd, 87.57.NK

## I. INTRODUCTION

Many modern linear accelerators are equipped with electronic portal imaging devices (EPIDs) for on‐ or off‐line patient or target repositioning and, hopefully, improved treatment accuracy. The efficacy of the EPID for this application is limited by poor image quality^(^ ^1^ ^,^ ^2^ ^)^ (low contrast, high noise), by lack of mechanical stability and reproducibility of the components, and by spatial distortion in the imaging chain of video‐based EPIDs.

Algorithms such as adaptive histogram equalization enhancement^(3)^ and wavelet‐based techniques^(^ ^4^ ^,^ ^5^ ^)^ can be used to enhance contrast by allowing various features in the portal image to be extracted and identified for positional analysis. Image noise reduction is often achieved through frame averaging^(6)^ or application of various filters, including subtraction and division.^(7)^ Spatial image distortion arising from the image chain can be corrected by a simple affine transformation. The affine transform, determined as part of routine quality assurance of the imager, is defined as a mapping of the projection of a known grid from real space into image space.

Many portal image applications and various clinical studies attempt to differentiate between the geometric effects of organ or target motion, patient setup error, and machine alignment errors [room lasers, optical distance indicator, multileaf collimator (MLC) or jaw calibration].^(^ ^8^ ^–^ ^13^ ^)^ Starting from a portal image, machine errors and setup errors can be distinguished only if an absolute reference point such as the intersection of the collimator axis of rotation with the image plane can be determined on each image. Many EPIDs are deployed only when images are required, and the EPIDs are susceptible to mechanical instability such that their position, with respect to the beam axis, can vary significantly with gantry angle and from day to day.

For our Siemens KD Mevatron (Siemens Oncology, Concord, CA), we have measured variations greater than 10 mm for the point (pixel) at which the collimator axis of rotation intersects the image plane of the TI EPID. The magnitude and direction of this variation depends on gantry angle, but is not reproducible from deployment to deployment. Several methods of correcting for image placement resulting from this instability have been reported in the literature.^(^ ^9^ ^,^ ^14^ ^)^ The simplest method is to use small high‐Z markers (BBs) mounted in an acrylic tray and placed in the shielding tray holder of the linear accelerator (LINAC). This approach is inappropriate for our work, because the BBs often obscure the patient's fiducial markers, making it difficult to extract anatomic information from the EPID images, and, less importantly, the BBs cause minor dosimetric anomalies.^(15)^ In other methods, field edges are extracted through global thresholding, and these are then compared or correlated with the planned field to determine the change in translation and orientation from planned to treatment geometry. The accuracy of these methods is compromised by MLC and jaw positioning errors alike and by EPID performance such as intensity uniformity. More importantly, all of these methods lack an absolute reference position, making it impossible to use the EPID to determine individual jaw and leaf calibration with respect to collimator axis of rotation for machine quality assurance or to distinguish positional errors in patient setup from leaf or jaw calibration errors. Notably, the field edge detection method can work very well if the vendor's specification on the jaw and MLC positioning accuracy are very tight and if they are maintained by using a rigorous quality assurance program.

Here, we present a method for accurately determining the intersection of the collimator axis of rotation with the EPID image plane, making geometric treatment analysis more robust. The approach is based on an inherent property of MLCs. Adjacent leaves of MLCs have a small gap between them, allowing a measurable amount of radiation (up to 2%) to be transmitted.^(16)^ When patients are imaged during radiation therapy, this interleaf leakage is integral to the portal image. Using image processing tools such as the Radon transform and cross‐correlation, the interleaf leakage can be extracted and used to accurately determine the spatial coordinates of imaged objects with respect to the beam isocenter. Having accurately and independently determined an absolute reference position, we can use local rather than global thresholding to more accurately determine leaf and jaw positions.

## II. METHODS AND MATERIALS

### A. LINAC, MLC, and portal imager

The present study was based on a Siemens Digital Mevatron LINAC equipped with a 58‐leaf double‐focused MLC (Siemens Oncology Systems, Concord, CA) and a Beamview TI electronic portal imager. The LINAC produces X‐ray photons with nominal energies of 6 MV and 18 MV. The imager consists of a gadolinium oxysulfide screen viewed through a mirror by a video camera and lens system placed at 90 degrees. The imager has a field of view of 26.5×24.8 cm projected to the plane of the isocenter and a square pixel dimension of 0.518 mm per side. The Siemens MLC consists of two opposed banks (X1 and X2) of 29 leaves each, defining the collimated field in one direction. The central 27 leaves of each bank project to exactly 1 cm width in the plane of the isocenter; the two outer leaves of each bank project to a width of 6.5 cm. A signature of the MLC is the interleaf leakage, which in the Siemens LINAC is about 1.5% of the central‐axis open‐field dose. The machining tolerance of the MLC leaves is very tight, resulting in a highly accurate and reproducible spacing of 10.0±0.1 mm of the interleaf leakage pattern at the isocenter. The positional accuracy of the MLC in its direction of travel has a specification of ±2 mm at isocenter, which is a principal cause for uncertainty in using field‐matching techniques to determine beam isocenter. In the direction perpendicular to leaf travel, the field edge is defined by solid tungsten jaws with a specified positional accuracy of ±2 mm.

### B. Segmentation of the portal image

**Figure 1:**
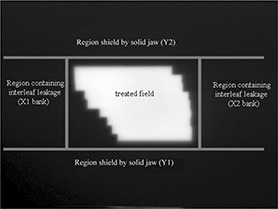
A typical clinical electronic portal imaging device image showing the region containing patient information (treated field) and the region containing spatial position information (region of interleaf leakage)

For the present work, we segmented the portal image into three zones as shown in Fig. 1:

- The open field portal (treated field)
- The area shielded only by the MLC leaves
- The area shielded by the solid jaws

Two segments, one from each of the regions shielded only by the two MLC banks, were selected for analysis. The length of the selected region was limited in the Y direction by the field size defined by the solid jaws. The width of the region was selected to be about 5 cm. The region was always at least 0.5 cm from the open field. These two regions were analyzed for interleaf leakage.

### C. The Radon transform

The Radon transform has been widely used in image reconstruction from projections for imaging modalities such as computed tomography and magnetic resonance.^(^ ^17^ ^)^ Recently it has been used for image processing—particularly for pattern recognition—because of its ability to transform line patterns in the image domain into dot patterns in the Radon domain. A domain of possible line parameters can be easily determined.^(^ ^18^ ^)^

A Radon transform (the ray‐integral of an image *f* along direction θ) is defined as

(1) $$g_{\theta }(r)=\iint f(x,y)\delta (r-x\cos\theta -y\sin\theta )dxdy,$$

where δ is the Dirac delta function, *f*(*x,y*) is the image intensity at location *x,y*, and r=xcosθ+ysinθ is the perpendicular distance from the image origin (image center) to the straight line along which the ray‐integral is evaluated. Equation 1 can also be regarded as the linear intensity integration of image *f* along a particular angle θ.

Equation 1 treats the image as a continuous function; but, in practice, a discrete Radon transform is used, as is nearest‐neighbor image interpolation for improved spatial resolution. Note that, because the integrated points are not always obtained from the original image matrix, the neighbor interpolation allows the interpixel intensity calculation that eventually improves the spatial resolution.^(18)^

When appropriately applied, the Radon transform is able to identify and extract weak lines embedded in very noisy images. In the present work, we used this property to extract interleaf radiation leakage from the background noise in the portion of clinical portal images shielded only by the MLC leaves. Note that, the lower the contrast and the noisier the image, the longer the integration line must be for accurate analysis.

**Figure 2:**
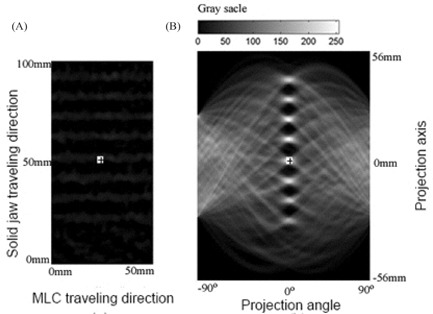
(a) Portal image containing interleaf leakage, and (b) the corresponding sinographic plot from the Radon transform, where MLC is multileaf collimator

Fig. 2(a) is a part of a clinical portal image cropped from the zone behind the X1 leaf bank containing interleaf leakage as described earlier. Fig. 2(b) is the corresponding sinographic plot of the Radon transform for that region. The horizontal axis represents the projection angle, and the vertical axis represents the distance of the ray‐integral from the image center. The origin of the coordinate in the image and Radon transform domains is marked with a “+” in Figs. 2(a) and 2(b) respectively. The leakage pattern is very faint and hardly discernable by eye in the original image. However, as can be seen, the Radon transform produces intense signals (spots) representative of the orientation and location of the interleaf leakage in Radon parametric space. Before the Radon transform was applied, the image was convolved with the Mexican Hat Function kernel^(^ ^4^ ^,^ ^5^ ^)^ to substantially improve the contrast of the interleaf leakage.

### D. Determination of orientation

**Figure 3:**
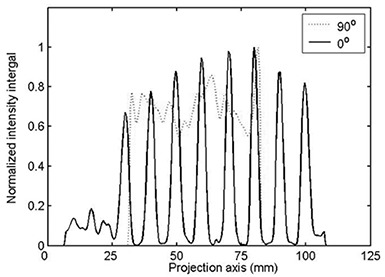
Radon transform of interleaf leakage parallel (0 degrees) and perpendicular (90 degrees) to the line of leakage

Fig. 3 shows the Radon transform $g_{\theta }(r)$ of the image *f* at 0 degrees (parallel) and 90 degrees (perpendicular) to the direction of motion of the MLC leaves.

The Radon transform can be considered the sum of all intensity values along the integration path. Thus, when the path of integration is parallel and coincident with the leakage radiation, the signal (sum) will be significant. When the integration path is parallel, but not coincident, with the leakage radiation or when the integration path is perpendicular to the leakage radiation, the signal will be weak. The expression $g_{\theta }(r)$ can be described as a stationary signal, modulated by the interleaf radiation leakage, with a period that is the width of a single leaf along the line of integration at angle θ. The signal contrast of interleaf radiation leakage can be defined as

(2) $$C(\theta )=\frac{100\times (s_{\theta }^{\max}-s_{\theta }^{\min})}{2s_{\theta }^{\max}},$$

where $s_{\theta }^{\max}$ and $s_{\theta }^{\min}$ are the maximum and minimum values of the signal $g_{\theta }(r)$ at angle θ. Thus, the signal contrast as a function of integration angle θ can be used as a means of determining leaf (and hence collimator) orientation in a portal image such that *C*(θ) is a maximum when the integration angle θ is parallel to the leaf orientation $\theta_{\text{leaf}}$ in the image. Then, if the collimator angle $\theta_{\text{col}}$ is well known, the relative rotation of the image chain with respect to the collimator rotation will be Δθ where

(3) $$\Delta \theta =\theta_{leaf}-\theta_{col}.$$

We tested the Radon transform method of determining collimator rotation with respect to the EPID grid for five relative angles (0, 1, 2, 3, and 4 degrees). The angle between the collimator rotation and the EPID grid was determined independently using the projected image of a machined grid mounted in the collimator's accessory holder. The grid lines were defined with respect to the solid Y‐jaw of the collimator to better than 0.1 degree. A precision level was used to define the collimator rotation at gantry 90 degrees, accurate to better than 0.01 degree. Images were acquired with 10–monitor unit (MU) exposures at 18 MV. For each case, the interleaf radiation contrast signal was determined as a function of projection angle θ. The method was tested for thee different cases:

- Using a single image frame to analyze the interleaf leakage behind a single‐leaf bank
- Using a single image frame to analyze the interleaf leakage behind both leaf banks
- Using a composite image made from six frames to analyze the interleaf leakage behind both leaf banks

### E. Determination of the beam isocenter

The Radon transform analysis of the interleaf leakage can be used to accurately determine the Y‐component of the intersection of the beam axis with the portal imager. The algorithm is described in this way: The Radon transform $g_{0}(r)$ parallel to the MLC leaves produces a high‐contrast signal, with peaks $\left(y_{i}^{peak}\right)$ at locations where the interleaf leakage is maximum. This signal will have a period of distance *d*, the projection width of an individual leaf. Assuming very high manufacturing tolerance, the Y component of beam isocenter $y_{\text{iso}}$ should be exactly in the middle of the 15th leaf for a 58‐leaf Siemens MLC. Locations for $y_{i}^{peak}$ are determined by

(4) $$y_{i}^{peak}=\{\begin{array}{ll} y_{iso}+(i-15.5)d & i>15\\ y_{iso}+(i-14.5)d & i<15\;, \end{array}$$

where *i* is the leaf index. A least‐squares fit of the measured $y_{i}^{peak}$ values to Equation 4 provides an accurate estimate of $y_{\text{iso}}$.

The position of the isocenter in the orthogonal direction can be determined by several methods. The most accurate and simplest is to rotate the collimator by 90 degrees and repeat the preceding method. But rotation is not always practical, particularly in the clinical environment. An alternative method uses local thresholding: Given that $y_{\text{iso}}$ is predetermined from the interleaf radiation leakage, a 5‐mm‐wide strip centered on each leaf *i* is segmented from the portal image. For each extracted strip *i*, the actual measured leaf edges $\left(x_{i,1}^{m},x_{i,2}^{m}\right)$ in the left and right banks are independently determined through local thresholding. The expected edge position $x_{i,j}^{e}$ of leaf *i* in bank *j* can be determined as

(5) $$x_{i,1}^{e}=x_{iso}+d_{i,1}^{p}$$

and

(6) $$x_{i,2}^{e}=x_{iso}+d_{i,2}^{p},$$

where the leaf positions $d_{i,1}^{p}$ and $d_{i,2}^{p}$ in two banks are assigned during planning. If the nature of the MLC positioning error is random, then $x_{\text{iso}}$ can be determined by minimizing the variance for the selected leaves, where

(7) $$ɛ=\sum_{i}\left\lfloor {\left(x_{iso}-d_{i,1}^{p}-x_{i,1}^{m}\right)}^{2}+{\left(x_{iso}+d_{i,2}^{p}-x_{i,2}^{m}\right)}^{2}\right\rfloor .$$

The advantage of local thresholding is that it eliminates the error that arises when global thresholding is applied to images that have intensity gradients across the field, because global thresholding uses a single threshold value to determine the beam edge (e.g., 50% dose). But, because of the intensity gradients, this global value may not be valid locally. The intensity gradients arise from patient attenuation and, in the case of our Siemens TI imager, from spatial variation in imager response.

We tested the ability of our method to determine the exact position of the intersection of the collimator rotation axis with the image plane for 22 different MLC‐shaped fields. All images were exposed to 10 MUs of 18‐MV photon beams. Four images were taken at each of the nominal oblique gantry angles (45, 135, 225, and 315 degrees) and six images were taken at gantry 0 degrees. The position of $y_{\text{iso}}$ was determined directly from the Radon transform as described earlier; $x_{\text{iso}}$ was determined from local thresholding and residual minimization as described earlier. The reference “true” isocenter position was determined under collimator rotation as the centre of the locus of projections of a 0.5‐mm steel sphere embedded in a plastic plate placed in the collimator accessory holder. For comparison purposes, the isocenter position was also calculated by the method of moments^(^ ^14^ ^,^ ^9^ ^)^ and the normalized cross‐correlation method.^(19)^

## III. RESULTS

**Figure 4:**
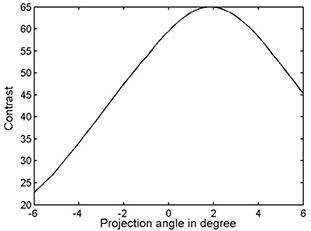
Contrast of interleaf leakage as a function of projection angle

Fig. 4 shows a typical *C*(θ), where the angle between the collimator and the EPID grid was known *a priori* to be 2.0±0.1 degrees. Table 1 shows the results of the collimator rotation test, where collimator orientation with respect to the image grid can be seen to agree to much better than 0.5 degree, exceeding the manufacture's specification for accuracy of collimator rotation. Overall, we are able to determine the collimator rotation to within 0.12±0.18 degrees, 0.12±0.11 degrees, and 0.08±0.11 degrees by analyzing the interleaf radiation leakage under both banks from a single frame, from double frames, or from a six‐consecutive‐frame average, respectively.

**Table 1:** Comparison of various measures for interleaf leakage analysis

Collimator angle ($\theta_{\text{col}}$, degrees)	Leaf orientation in portal image ($\theta_{\text{leaf}}$, degrees)		
	X1 bank	Double bank	Frame averaging
0.0	0.2	0.0	0.2
1.0	1.0	1.2	1.0
2.0	2.0	2.2	2.2
3.0	3.0	3.2	3.0
4.0	4.4	4.0	4.0

Table 2 gives the results for the exact position of the intersection of the collimator rotation axis with the image plane, showing mean and standard deviation of the difference from the true isocenter for each of the three methods. The method presented here can be seen to provide a better estimate of isocenter in the direction perpendicular to the interleaf leakage. In the direction of MLC leaf travel, the method of moments provides a better estimate.

**Table 2:** Deviations from true isocenter for three methods

Method	Deviations (mm)	
	X	Y
Leakage	0.77±0.31	0.63±0.25
Correlation	0.97±0.36	0.85±0.37
Moment	0.72±0.36	1.07±0.65

## IV. DISCUSSION AND CONCLUSIONS

In our experience, the mechanical instability of our portal imagers has acted as a barrier to fully exploiting portal images for LINAC quality assurance and for accurate distinction between patient setup errors and MLC leaf and jaw calibration errors. In the present work, we investigated the feasibility of using interleaf radiation leakage to accurately determine collimator rotation. We used the interleaf leakage to accurately estimate the position of the beam isocenter perpendicular to leaf travel in the EPID image and minimization of residuals, aided by local thresholding, to estimate the isocenter in the leaf travel direction. We showed that the Radon transform can successfully extract position and orientation of interleaf leakage from the electronic portal image and that this information can be used to accurately determine the collimator rotation.

Electronic portal imagers could be ideal devices for filmless quantity assurance of treatment machines, and particularly for MLC and collimator calibration; but they are not being used for this purpose in most radiotherapy practices. A recent report^(20)^ indicated that, at the end of 2002, only 5% of U.S. cancer centers employed electronic portal imagers for filmless quality assurance. Among the barriers to implementation are poor image quality and mechanical instability. We believe that the methods presented here can, in part, address many of those concerns.

The efficacy of portal imagers as a tool for image‐guided radiotherapy is also limited by their performance—particularly their lack of mechanical stability and reproducibility (orientation and translation) from field to field and fraction to fraction, and their low image contrast and the geometric distortion inherit in some video‐based systems.

We demonstrated a method that can overcome the issue of mechanical instability and that can provide an accurate estimate of position (e.g., the intersection of the collimator axis of rotation with the image plane). Armed with this information, use of an EPID for routine MLC and jaw quality assurance can be considered. Given a single portal image, information about the location of the collimator axis of rotation makes it possible to distinguish between the geometric errors arising from patient setup, collimator rotation calibration, and the random geometric errors in positioning MLC leaves. We are currently using this approach to analyze EPID images from a large series of prostate patients, accurately quantifying interfraction setup errors, intrafraction patient motion, and treatment‐specific daily errors in MLC calibration.
